# Mucormycosis: A comparative update between conventional and molecular diagnosis strategies

**DOI:** 10.18502/cmm.8.1.9214

**Published:** 2022-03

**Authors:** Shikha Mudgal, Shalinee Rao, Manju O Pai

**Affiliations:** 1 Department of Pathology and Lab Medicine, All India Institute of Medical Sciences, Rishikesh, Uttarakhand, India; 2 Department of Pathology and Lab Medicine, Division of Molecular Biology, Metabolomics and Proteomics, All India Institute of Medical Sciences, Rishikesh, Uttarakhand, India; 3 Division of Microbiology (DRDO-RJSR), All India Institute of Medical Sciences, Rishikesh, Uttarakhand, India

**Keywords:** Angio-invasive, *Aspergillus*, Molecular methods, *Mucor*, Mucormycosis, PCR, Rhizopus, Serum, Urine

## Abstract

Mucormycosis is an opportunistic, aggressive, and angioinvasive fungal infection associated with a high mortality rate as it disseminates and infects the whole body if not treated early. Most conventional diagnostic methods require time and may also generate false-negative reports due to the several lacunae associated. On the other hand, molecular methods are rapid, reliable, and can be applied to different biological samples, such as fresh tissue, formalin-fixed paraffin-embedded blocks, serum, and urine. Mucorales are angio-invasive, and many studies have found the circulating fungal DNA (a non-invasive form of DNA) in the blood and urine of the patient. In addition, with the increase in the usage of steroid drugs in this COVID scenario, the rate of mucormycosis infection has taken a sudden rise. In light of this situation, there is an imperative need to diagnose these infections at the earliest

## Introduction

Mucormycosis is an opportunistic fungal infection caused by species of order Mucorales, phylum Zygomycota like *Rhizopus*, *Mucor*, *Lichtheimia*, *Rhizomucor*, *Saksenaea*, *Cunninghamella*, and *Apophysomyces* [ 1 ]. Both modes of reproduction (i.e. sexual and asexual) are present in Mucoralean fungi. Most pathogenic Mucorales are heterothallic and reproduce sexually to produce zygospores, which is a time-consuming process as viewed by Mendoza et al. [ 2 ]. On the other hand, asexual sporangiospores produced by sporangiophores disperse and germinate rapidly to form a mycelial complex composed of ribbon-shaped aseptate hyphae. Sporangiospores so formed are the major source of airborne infection [ 3 ]. 

Mucormycosis has different clinical manifestations. These forms can be classified based on the disease severity and localization into Rhino-orbito-cerebral, cutaneous and soft tissue, pulmonary dissemination, gastrointestinal, and renal mucormycosis [ 1 ]. The most common route of infection is the inhalation of sporangiospores bigger than 10 micrometers. These spores localize/ colonize in the upper respiratory tract and cause sinus or rhino infection. Fungal colonization in the distal respiratory tract is a source of pulmonary infection. If not treated on time, the rhino-orbital form may spread to the cerebral regions of the brain and cause rhino-cerebral mucormycosis or can even disseminate to other parts of the body [ 3 ]. Physical trauma caused by burn or accident damages skin as the first line of defense and makes patients more vulnerable to cutaneous and soft tissue mucormycosis. Weakening of immune response and neutropenia occurs in patients with hematological malignancies, such as acute lymphocytic leukemia, acute myeloid leukemia, chronic myeloid leukemia, and bone marrow transplant recipients undergoing chemotherapy or any kind of radiation therapy or chemotherapy. These modalities in patients make them susceptible to mucormycosis [ 4 ]. 

Patients with diabetes mellitus, and recurring diabetic ketoacidosis are prone to all forms of infectious mucormycosis [ 5 ]. Diabetes causes impaired host defense and weakens neutrophils’ ability to adhere to endothelial walls and do phagocytosis. On the other hand, diabetic ketoacidosis causes hyperglycemia and an increase in the availability of micronutrients, such as iron. Moreover, it reduces the blood pH and impairs serum's ability to bind iron, creating a perfect environment for fungal invasion [ 6 ]. Fungal hyphae produce rhizoferrin, which binds vividly to iron for fungal invasion (Rhizoferrin- iron complex is used for the fungal intracellular process). Human body resistance to fungal invasion is developed through the reduction of available iron by the facilitation of excess iron binding to a protein called apotransferrin [ 7 ]. In developing nations, poor nutrition or malnutrition causes a defective immune system which in turn increases the risk of fungal infection. In developed countries, this disease is commonly diagnosed in patients with hematological malignancies and transplant recipients whose immune system is compromised [ 8 ]. In cases where mucormycosis is not timely diagnosed and treated, the fungus keeps multiplying and produces more sporangiospores, which results in the secretion of several toxins and proteases that destroy the endothelial cells in the mucosal membrane as well as angioinvasion and further spread of infection.

An alarming hike in mucormycosis cases was reported around the globe during the second phase of COVID-19 [ 9 ]. Similarly, India has witnessed an alarming rise in mucormycosis cases during the COVID-19 pandemic [ 10 - 14 ], and COVID-19 patients reported a high incidence of secondary bacterial and fungal infections, possibly due to compromised immunity. One hypothesis is that the extensive use of therapeutic agents (e.g., steroids, monoclonal antibodies, or broad-spectrum antibiotics) to combat SARS CoV-2 infection can elevate the risk of fungal infections [ 10 ].

### Conventional diagnostic methods for identification of mucormycosis

The mortality rate of mucormycosis is high; therefore, any delay in the diagnosis and management can be life-threatening. Direct examination of the infected specimen under the microscope or through culture is advised for an irrefutable diagnosis.

### Examination of the clinical specimen under the microscope

Cytological (e.g., sputum and fluid aspirate from the site of inflammation) and tissue biopsy samples are diagnosed for Zygomycetes, with characteristic ribbon-like aseptate, broad and branched (generally at 900) hyphae. The most commonly used stains for cytological examination include Papanicolaou stain, Gram’s stain, periodic acid-Schiff, calcofluor white stain, Gomori’s methenamine silver stain, Fungi-Fluor, and Hematoxylin and Eosin (H&E). However, the major drawback associated with Zygomycetes direct microscopic diagnosis is the presence of rare and fragmented fungal elements in the cytologic samples, as the extraction of the specimen from the site of infection might not be possible. The other factors observed low or inconsistent staining intensity of Mucorales sample, and sometimes tissue gets folded during the processing of biopsy samples, which might lead to misdiagnosis or the false-negative finding of the histopathology or cytology microscopic examination [ 15 , 16 ]. A total of twenty-six (12 males with a mean age of 51±12.06 years and 14 females with 55.17±11.7 years of age) mucormycosis patients were diagnosed in 2019-2020. At our centre, all the histopathology mucormycosis diagnoses reported during 2019 -2020 were analysed within 2-7 days (Table 1).

**Table 1:** Diagnosis time of AIIMS Rishikesh Mucormycosis cases (2019-2020) compared with culture reports

Specimen	Age/Male	Net reporting time in days	Histopathology Diagnosis	Culture Diagnosis
Left middle meatus	22/M	2	Features are suggestive of mucormycosis	Broad aseptate fungal hyphae observed
Necrotic bone from right maxilla; Granulation tissue around the necrotic bone	63/F	4	Features of mucormycosis with bacterial colonies	NA
Nasal cavity	51/M	4	Mucormycosis	Candida glabrata isolated in culture. Significance to be correlated
Edge of the ulcer	46/M	2	Mucormycosis	No fungal growth obtained after aerobic incubation for 4 weeks.
Hard palate	57/M	10	Mucormycosis	No fungal elements observed
Left nostril mucosal tissue	45/F	4	Mucormycosis	Rhizopus and Alternaria spp. isolated in culture after 3 days of aerobic incubation.
Sinonasal debridement tissue	45/F	3	Mucormycosis	NA
Nasal cavity	60/F	7	Features suggestive of invasive mucormycosis	No fungal elements observed
Right sphenoid sinus mucosa and right middle meatus mucosa	65/M	2	Biopsy of right sphenoid sinus mucosa- Angioinvasive Mucormycosis	Rhizopus arrhizus and Aspergillus fumigatus and Aspergillus flavus isolated after 21 days of aerobic incubation at 37°C.
Biopsy from the nasal cavity	60/F	7	Invasive mucormycosis	No fungal elements observed
Otitis extent.	65/F	5	Possibility of aseptate fungi, possibly mucormycosis	Broad, aseptate hyphae observed
Left nasal cavity	35/M	4	Showing fungal organism mucormycosis	No fungal elements observed
Chronic invasive fungal sinusitis	61/F	2	Suggestive of Mucormycosis	Hyaline septate fungal hyphae observed
				*Aspergillus flavus* isolated in culture after aerobic incubation at 25 and 37º celsius.
Debris from right mucosal sinus	27/M	7	Fungal rhinosinusitis, morphologically suggestive of mucormycosis	Plenty of septate fungal hyphae with acute angle branching conidial head suggestive of Aspergillus spp.
Superstructural maxillectomy; Left cheek muscle with subcutaneous tissue with skin; Left eye (orbital exenteration) and excised nasal septum	49/F		Invasive mucormycosis	Broad aseptate hyaline fungal hyphae observed
Necrosed tissue from the middle turbinate	41/F	5	Suggestive of mucormycosis	No fungus isolated in the culture
Nasal cavity near middle turbinate	35/F		Consistent with Mucormycosis	Rhizopus spp. isolated in culture
Palate	54/M	6	Features are of mucormycosis	Rhizopus species isolated in culture
Nasal cavity	15/F	7	Features compatible with mucormycosis	No fungus isolated in the culture
Left nasal cavity	31/M	10	Angioinvasive mucormycosis	Predominantly broad aseptate fungal hyphae with sparse hyaline septate hyphae observed
Middle meatus mucosa	65/F	8	Features of mucormycosis	Plenty of broad aseptate hyphae observed
Middle ear mucosa; Sloughed cartilage areas	9/F	4	Middle ear mucosa: mucormycosis	NA
Sinus and nasal cavity	42/M	4	Invasive mucormycosis	NA
Right eyelid, Right nasal turbinate with nasal mucosa and maxillary bone	42/M	3	Invasive fungal sinusitis consistent with mucormycosis	No fungal elements observed
Left middle turbinate region	73/F	7	Features are consistent with nasal mucormycosis with tissue invasion	NA
Necrotic tissue from the right maxilla	58/M		Features are suggestive of mucormycosis, tissue invasive	Broad aseptate perpendicular branching ribbon-like hyphae suggestive of mucormycosis
Palate and hard palate	74/M		Mucormycosis	NA

### Culture examination of Mucorales

Commonly used media for the growth of Mucorales include corn meal agar, potato dextrose agar, potato carrot agar, beef extract agar, malt extract agar, oatmeal agar, glucose yeast extract, inhibitory mold agar, and Sabouraud dextrose agar. The specimen was cultured and incubated at 30ºC or 37 ºC for 3-5 days in a clinical microbiology lab. Mucorales (e.g., *Mucor* sp.) have different growth temperatures, and the best growth has been obtained at <37ºC. The best growth temperature for *Rhizomucor* and *Rhizopus* sp is 20-60 ºC QUOTE ºC and 37 ºC, respectively [ 17 ]. Mucorales tends to cover the entire plate with characteristic areal hyphae which create a “cottony” like appearance, and coloration can also be seen from the reverse side of the Petri plate for 5-7 days. After growth, the specimen is examined under the microscope for further characterization using different techniques for isolating zygomycetes from the culture plate on the slide, including tease prep, tape prep, slide culture, or lactophenol cotton blue (LPCB) staining. The varying growth rate of the mucor, height reached by aerial mycelium, and degree of coloration during sporulation were different between species of Mucorales and are used for species identification [ 16 ].

Characterization and classification of Mucorales into the family, genus, and species were performed based on asexual spore-producing structures, such as (i) Sporangium, (ii) Sporangiole, (iii) Merosporangiun (iv), and sexually produced zygospore. Mucorales, such as *Rhizopus*, *Rhizomucor*, *Mucor*, *Absidia*, *Mortierella*, *Apophysomyces*, and *Saksenaea* produce asexual spores called sporangiospores on a sac-like structure called sporangia, which are classified as sporangium producers. *Cunninghamella Bertholletia* and *Cokeromyces recurvatus* acquired swollen vesicles called sporangia producing single-cell asexual spores. 

The only Mucorale species, *Syncephalastrum racemosum*, produces merosporangium (spores formed in the tubular sac around swollen vesicle). Zygospores are either homothallic or heterothallic (reproduced with or without mating). The homothallic reproduction is characteristic of a few members of Mucorales and is observed in *Rhizomucor miehei* [ 17 ] and *Cokeromyces recurvatus* [ 18 ]. Most Mucorales are heterothallic; therefore, zygospores are missing and identified by the morphology of the asexual spores [ 16 ].

Microbiology culture methods play an important role in the diagnosis of mucormycosis; however, the technique is labor-intensive and requires a lot of expertise for the isolation and identification of the causative agent [ 11 ]. On the other hand, the time of diagnosis is approximately 7 to 8 days, causing a delay in treatment which could be detrimental and even lead to death. Kontoyiannis et al. [ 19 ] reported low sensitivity and inefficiency of culture techniques in isolating zygomycetes from the tissue specimen. At our center, all the histopathology mucormycosis reports during 2019-2020 were compared with the culture reports to find the agreement rate between the two techniques. After the elimination of six cases due to the unavailability of culture reports, it was revealed that 36% (8/11) of reports were in agreement (Table 1). 

It should be noted that there is no standard test for the identification and diagnosis of mucormycosis, and the correct identification and classification have become the bottleneck of research on Mucorales. Several molecular techniques have been tested for the identification and classification of fungus.

### Molecular Methods

All the conventional methods of Mucorales diagnosis are morphology-based and prone to high error rates. Many chemical and molecular characters, including sterols, fatty acids, protein, and deoxyribonucleic acid-based methods were tried and tested for a fast and reliable diagnosis. Weete and Gandhi (1997) [ 20 ] found the presence of different sterol compositions in the Zygomycetes. Koch and Botha (1998) [ 21 ] and Frisvad et al. (2008) [ 22 ] tested fatty acids. Many serology-based detection methods use ELISA for the detection of antibodies produced against Mucorales infection [ 23 , 24 ]. To date, the most accurate and successful method has been DNA barcoding which was identified in the level of species in the most neglected taxa. DNA barcode is a short DNA region known for taxonomic classification of fungus and is used extensively in the identification of fungal species [ 25 , 26 ].

### Sequencing and Polymerase Chain Reaction

The use of molecular techniques has promoted the accurate and timely identification of Mucorales fungi. The first molecular diagnosis through Polymerase Chain Reaction (PCR) was performed using tissue and bronchoalveolar lavage in immunocompromised patients admitted to the intensive care unit in 2013 [ 26 ]. Mucormycosis patients’ blood, formalin-fixed paraffin-embedded (FFPE) and tissue samples have been tested by PCR using different primers by scientists in their research. Mucorales are angioinvasive in nature and several studies have found the presence of circulating DNA of the fungus in patients’ blood and urine samples, indicating that PCR can be considered a non-invasive technique for the identification and classification of Mucorales. Several other advantages of PCR include [i] small amount of DNA [1 to 0.1 ng] needed for amplification from blood and urine samples [ii], amplification of both forward and reverse DNA strands which reduce the error rate, and [iii] short delivery time of test results (within 3-4h) [ 27 ] which expedites the administration of treatment. Many molecular techniques had been used so far using either a single gene, DNA barcode, or whole genome.

### Different primers or DNA barcodes

Both 18S and 28S nuclear r RNA genes have evolved slowly, compared to mitochondrial r RNA gene and internal transcribed spacer (ITS) (used extensively for Mucorales diagnosis), making them more suitable for studying distantly related organisms. Moreover, 18S ribosomal RNA is divided into V1 to V9 regions that contain both conserved and hypervariable regions. Most universal 18S r RNA primers are designed against V2, V4, and V9 regions, enabling family and order level identification of fungal species [ 28 ]. 18S r DNA has been proved useful for the identification of fungal phyla *Saccharomycotina*, *Taphrinomycotina*, *Pezizomycotina*, *Mucoromycotina*, *Pucciniomycotina*, and *Agaricomycotina*. Semi nested PCR on V4 and V5 regions of 18S ribosomal DNA for the identification of Mucorales by Gandhi et al. showed a high concordance rate of 100% in PCR and histopathology results [ 29 ]. Rickerts et al. [ 30 ] confirmed the diagnosis of zygomycosis in a case report of a 33-year male using panfungal PCR on the 18S gene, and the causative agent identified included *Cunninghamella bertholletiae*, *Absidia glauca*, *Cunninghamella elegans*, and *Cunninghamella polymorpha*. Kobayashi et al. [ 31 ] reported the confirmed diagnosis of a 54-year female with pulmonary mucormycosis caused by *Cunninghamella bertholletiae* using panfungal PCR assay on 28S DNA. Wu et al. [ 32 ] developed an assay by sequencing 18S r RNA for the identification and classification of different airborne pathogenic fungal strains. 18S region is less variable along with the kingdom fungus which limits its use in taxonomic resolution. Zhao et al. [ 33 ] addressed the shortcoming of 18S as the most conserved and inadequate molecular marker for reliable species identification.

Internal transcribed spacer, ITS1, and ITS2 are used for phylogenetic analysis. ITS1 and ITS2 are present between 18S, 5.8S, 5.8S, and 28S r RNA cistron. This region is also called standard fungal barcode since the sequence varies between different fungal species but is conserved among the same fungal species. ITS is superior to 18S for the identification of genera *Aspergillus* and *Candida*, however, the observed heterogeneity in Mucorales in the ITS region has occurred due to insertion and deletions that lead to inaccurate identification and classification of species. On tissue samples, pan fungal PCR targeting the ITS region (ITS1- 5.8S- ITS2) resulted in the successful identification of eight fungal pathogens [ 34 ].

Wagner et al. performed a study on 233 retrospective clinical samples and made a comparison between culture, ITS PCR, and 18S r DNA PCR. They found a concordance rate of 91% between culture results and 18S ribosomal DNA real-time PCR results and an additional 94% agreement between ITS PCR and 18S RT- PCR results. In clinically confirmed fungal infection cases, Sanger’s sequencing and 18S ribosomal real-time PCR found fungal DNA, while culture and ITS PCR presented negative results [ 35 ]. Taurenne et al. (1999) reported the rapid and accurate speciation of many clinically important fungal species, including Mucorales, using ITS r DNA [ 36 ]. Nagao et al. (2005) used the ITS r RNA gene for the identification of pathogenic *Rhizopus* species via multiplex PCR [ 37 ]. ITS region is considered a DNA barcode since it is the most widely used molecular marker for phylogenetic characterization of fungal species [ 38 - 40 ]. Aanen et al. [ 41 ] reported a high degree of polymorphism in the ITS region which complicates the process of species identification. Eberhardt et al. used ITS molecular marker to identify species of genera *Hebeloma* and found multiple peaks as a signal due to unclear base calls raised as a result of many ITS copies in the genome [ 42 ].

Mitochondrial rnl gene (encoding the large subunit ribosomal RNA) [ 43 ] has revealed a sensitivity of 71.4%-78.9% in FFPE samples for identification of Mucorales which is higher than the postulated sensitivity (56% to 80%) of any assay format in FFPE sample. As *rnl* is a mitochondrial gene, it is less susceptible to necrotic degradation, which is characteristic of mucormycosis. This is an added advantage of mitochondrial genes over other merits, including high copy number per fungal cell and high stability due to the presence of a double membrane.

Circular mitochondrial cytochrome b (*cytb*) gene proved to be an efficient gene marker for the identification of species due to its remarkable variability between the species and lack of variability within the species [ 44 ]. Hata et al. (2008) performed real-time PCR to detect zygomycete genera species using primer and fluorescence resonance energy transfer hybridization probes against conserved 167 bp long zygomycete cytb gene. Mucorales genera *Absidia*, *Apophysomyces*, *Cunninghamella*, *Mucor*, *Rhizopus*, and *Saksenaea* were successfully detected using cytb molecular marker. The sensitivity and specificity of the PCR assay using cytb gene against culture were 100% and 92%, as observed by Hata et al. [ 45 ].

### FTR1 molecular marker

Iron is an important element for fungal metabolism and expression genes related to iron uptake are high in fungal pathogenic species, compared to non-pathogenic species. Four pathways of iron assimilation and metabolism in fungus reported in the studies conducted respectively by Lebreton et al. (2020) and Angelique et al. (2014) on *Mucor* species and *Aspergillus niger* include (i) iron uptake by protein siderophore, (ii) uptake and assimilation of heme iron via surface transporters of heme oxygenase family, (iii) low-affinity iron permease transporters (FET4) mediated iron uptake and assimilation taking iron and other metals, such as zinc and copper, and (iv) high affinity mediated iron permease (FTR1) transporter-mediated transport also called reductive iron assimilation (RIA) [ 46 , 47 ]. Nyilasi et al. [ 48 ] successfully tested and identified species of genera *Rhizopus*, *Rhizomucor*, and *Syncephalastrum* via FTR1 PCR amplification. PCR-restriction fragment length polymorphism (RFLP) of the FTR1 gene at the Alu1 restriction site has further identified subgroups of *Rhizopus oryzae*. 

Spore forming gene is a universally present spore coating encoding protein (*CotH*) in Mucorales that renders them pathogenic and helps in invasion as explained by Wolk et al. (2012). This study showed that the glucose-regulated protein 78 (GRP78), a host receptor on endothelial cells expression, elevates in hyperglycemia and Dia Ketoacidosis, and the spore-forming protein CotH3 serves as a fungal ligand for the protein; therefore, mediating fungal invasion [ 49 ]. Baldin et al. [ 50 ] investigated whether PCR amplified CotH gene can be tested in urine, plasma, and bronchoalveolar lavage (BAL) samples by infecting mice with different pathogenic Mucorales. They infected the mice intratracheally with different Mucorales (i.e., *Rhizopus delemar*, *Rhizopus oryzae*, *Mucor circinelloides*, *Lichtheimia corymbifera*, or *Cunninghamella bertholletiae*) and tested the plasma, urine, and BAL samples using CotH PCR assay. They recorded high sensitivity and specificity (90 and 100%) of the test and reported the authenticity of the CotH molecular marker in early and accurate diagnosis of mucormycosis.

Machouart et al. (2006) performed PCR-RFLP on FFPE samples for rapid identification of infection-causing Mucorales. Initially, DNA amplification was conducted for Mucorales species *Mucor*, *Rhizopus*, *Rhizomucor*, and *Absidia*. The restriction was applied using suitable restriction enzymes after amplification for species-level identification [ 51 ].

### PCR-Electrospray Ionization Mass Spectrometry

Genomic material (DNA or RNA) is extracted from isolated fungal colonies or biological samples and amplified by multiplex real-time PCR for a broad set of primers targeting the conserved area of multiple genes under stringent conditions. The amplicons are ionized by electrospray ionization and are subjected to mass spectrometry. The results so obtained are correlated to different databases.

In a study conducted by Alanio et al. [ 52 ], the diagnosis of mucormycosis was conducted by PCR/ESI-MS on tissue samples of 13 patients, and the results were compared with culture, 18S PCR sequencing, 16S-18S ribosomal RNA ITS PCR, and real-time PCR. The concordance rate of the culture result and PCR/ESI-MS results were high, compared to any other technique. Identification at the genus​ and species level was achieved and the results were out within 6 h of sample collection.

### Matrix-Assisted Laser Desorption Ionization- Time of Flight Mass Spectrometry

Matrix-Assisted Laser Desorption Ionization- Time of Flight Mass Spectrometry (MALDI- TOF-MS) is a high-throughput technique that generates a protein mass spectrum called “protein fingerprints” which are unique for species and allow for rapid, cost-efficient, and easy identification and classification of organisms. The presence of cell walls often complicates identification by MALDI- TOP MS; however, researchers overcame this problem by extracting intracellular proteins from fungal isolates [ 53 ].

Schwarz et al. (2011) has conducted a study to check the accuracy of MALDI- TOF MS in the identification of Mucorales species and have documented a high score of accuracy [ 54 ]. Shao et al. [ 55 ] also documented high accuracy and reliable results of MALDI-TOF MS in the diagnosis and classification of mucormycosis-causing fungi. Dolatabadi et al. [ 56 ] conducted a study to find whether MALDI -TOF MS could differentiate *Rhizopus* microspores from *R. arrhizus* and found that the technique could classify different varieties. They confirmed these findings by ITS sequencing and multilocus sequencing of ITS, ACT, and TEF gene markers. Vella et al. [ 57 ] identified disease-causing filamentous fungus [*Mucorales*, *Aspergillus*, and *Fusarium*] through the analysis of the surface antifungal susceptibility patterns of mature colonies to find fingerprints that are specific to species using MALDI-TOF MS, and the obtained results were in agreement with molecular analysis. Multiple studies have proved that MALDI TOF MS is a clinically relevant tool for the classification and identification of filamentous infectious fungal species.

### High-Resolution Melt curve analysis

High-Resolution Melt (HRM) curve analysis is a fast, powerful, ​and ​simple technique for the detection of changes in the double-stranded DNA molecule and is used to identify and classify fungal strains and mutations. This analysis is achieved through the binding of ds DNA to fluorescent intercalating dye, which produces high fluorescence when DNA is double-stranded and fades as the two DNA strands separate by the melting of DNA during PCR. This results in the production of curves with different fluorescent intensities which are used to study GC content, length, and sequence of amplicon [ 58 ].

Hrncirova et al. (2010) identified Mucorales species in tissue samples using PCR/ HRMA and concluded it to be an effective diagnostic procedure [ 59 ]. Lengerova et al. (2010) also used PCR/HRM analysis to identify Mucormycetes in Bronchoalveolar Lavage Fluid (BALF) and diagnosed the four most clinically relevant Mucorales in immunocompetent patients [ 60 ].

### Whole Genome Sequencing

Since 1977, mucormycosis outbreaks have been reported internationally [ 61 ]. This technique bridges the link between different cases with different strains of Mucorales that were identified from different areas of outbreaks and helped to understand the epidemiology of such outbreaks. A similar outbreak of invasive wound mucormycosis in a French hospital was done using Whole Genome Sequencing (WGS). This helped identify all the infection-causing strains and follow a timely and proper treatment for patients at the right time on a mass scale and led to a better understanding of transmission patterns [ 61 ].

### Advantages of Molecular Technique

Mucormycosis is a highly invasive fungal infection that needs an expedited diagnosis and treatment. Routine diagnostic procedures are time-consuming; however, this issue can be overcome by advanced molecular diagnostic procedures which generate results within a few hours of sample collection. Molecular methods are also more accurate and reliable than the standard diagnostic approaches used. These techniques can be used for different sample types, including FFPE, fresh tissue, BAL, urine, and serum (Table 2). It can also be used in non-invasive diagnosis if the samples included are sputum and urine (Table 3).

**Table 2:** Evaluation of different clinical samples: Different studies were conducted on different types of biological samples using molecular techniques and assays with different sensitivity and specificity

Author	Patients’ condition	Sample size	Sample type	Molecular technique	Assay	Sensitivity	Specificity	References
Springer 2016	Invasive mucormycosis	15	Serum	PCR	Muc18S assay	91%	NG	[ 63 ]
Millon 2016	Invasive mucormycosis	44	Frozen Serum	PCR	Muc18S assay	81%	NG	[ 64 ]
Zaman 2017	Rhino- orbito- cerebral	50	Fresh tissue	PCR- RFLP	Culture	48%	100%	[ 65 ]
					ITS2	54%	100%	
Legrand 2016	Invasive wound mucormycosis in critically burn patients	77	Plasma	qPCR	cm DNA	100%	NG	[ 66 ]
Lengerova 2014	Immunocompromised patients	86	BAL	PCR/ HRMA	ITS2	100%	93%	[ 61 ]
Springer 2018	Invasive pulmonary fungal infection	96	BALF	Real time PCR	18S and 28S rRNA	68%	93%	[ 67 ]
Scherer 2018	Pulmonary mucormycosis	374	BAL	qPCR	Muc18S assay	100%	97%	[ 68 ]
Baldin 2018	Mucormycosis	-	Urine	Real time PCR	CotH	90%	100%	[ 69 ]
Millon 2013	High risk patients	10	Serum	PCR	Hydrolysis probes	ng	NG	[ 70 ]
Hammond 2011	Hematologic malignancy	27	FFPE	PCR and sequencing	18S r RNA	81%	NG	[ 71 ]
Bialek 2005	Invasive fungal infection	52	FFPE	Seminested PCR assays	18S r RNA	59%	100%	[ 72 ]

FFPE: formalin-fixed paraffin-embedded tissue; BAL(F): Bronchoalveolar-lavages (Fluid); NG: not given.

### Disadvantages of Molecular techniques

Molecular markers or DNA barcode plays an important role in species identification; however, the process of species-specific marker identification is complex. The major problem in molecular marker design is the lack of standard, comprehensive, and reliable databases. Available web resources that are working continuously to overcome the problem of limited data include Barcode of Life Data Systems (www.boldsystems.org), MycoBank (www.mycobank.org), Global Biodiversity Information Faculty (www.gbif.org), Index Fungorum (www.indexfungorum.org), and GenBank (www.ncbi.nlm.nih.gov). Another challenge is the identification of appropriate controls (e. g., internal control and positive control) for more specific analysis for PCR-based assay and the quantity of DNA extracted from such samples as sputum and urine for PCR assay (Table 3).

**Table 3:** Advantages and disadvantages of molecular techniques

Advantages of molecular vs. conventional techniques	
Conventional techniques (Histopathology and Culture)	Molecular techniques
Histopathology sample analysis takes approximately 2 to 7 days and the culture method requires 48 to 72 h	Approximately 2- 6 h are required to process and analyze samples
Invasive	Non- invasive
Sample processing is labor-intensive	Non-labor intensive
Error during sample processing might lead to misinterpretation of results	Technical precision during DNA/RNA extraction
**Disadvantages of Molecular over Conventional techniques**	
Economical	Expensive
Well developed and established (histopathology is still considered a Gold Standard)	Insufficient literature and database
	Lack of reference standards and controls
	Delay in standardization of technique

## Conclusion

Characterization and identification of mucormycosis can be performed through macroscopic or microscopic methods. At our centre, histopathological diagnosis of mucormycosis takes approximately 2 to 7 days, and culture reports are out within 48 to 72 h, with a low agreement rate between the results. Conventional methods are time-consuming, labor-intensive, challenging, and require expertise. In addition, accurate identification of Mucorales species is vital for further treatment management. Molecular methods are valid, rapid, and non-invasiveness (due to application on urine, serum, and sputum samples); however, they hold limitations. Primers and assays used for molecular detection of Mucorales are not yet validated and clinically evaluated. Continuous research is going on worldwide to test the diagnostic analysis of different assays by measuring their sensitivity and specificity, which has yielded promising results.

The Fungal PCR Initiative (FPCRI, www.fpcri.eu) is working to include fungal PCR diagnostics to EORTC/MSG guidelines as successfully conducted for *Aspergillus* sp. In 2017 and 2018, in an inter-laboratory study, 23 European laboratories on two serum panels spiked with Mucorales species DNA, and the study results demonstrated good reproducibility and performance of the qPCR assay [ 62 ]. FPCRI/ Mucorales lab working group of the International Society for Human and Animal Mycology (ISHAM; https://www.isham.org/) is constantly working on the improvement and validation of the Mucorales-based qPCR assay.

## Acknowledgments

Mudgal S. acknowledges all authorities in All India Institute of Medical Sciences, Rishikesh, Uttarakhand, India, for the offered Ph.D. fellowship and their assistance during the writing of this manuscript.

## Authors’ contribution

SM wrote the manuscript. MOP designed, planned, and edited the manuscript, and SR reviewed it. All the authors commented on the manuscript. All the authors read and approved the manuscript.

## Conflicts of interest

The authors declare that they have no conflict of interest regarding the publication of the present study.

## Financial disclosure

The authors received no funds for the publication of this manuscript.
